# Sympathetic nerve activity in obesity-associated insulin resistance

**DOI:** 10.1016/j.apsb.2025.01.018

**Published:** 2025-01-28

**Authors:** Shuchou Xia, Huili Wu, Jianping Ye

**Affiliations:** a Institute of Trauma and Metabolism, Zhengzhou Central Hospital Affiliated to Zhengzhou University, Zhengzhou 450007, China; b Tianjian Laboratory of Advanced Biomedical Sciences, Academy of Medical Sciences, Zhengzhou University, Zhengzhou 450001, China

**Keywords:** Insulin resistance, Sympathetic nerve activity, Energy surplus, Obesity, Nutrition overload

Insulin resistance (IR) is a risk factor of type 2 diabetes and cardiovascular diseases^1^. IR is a result of energy surplus often associated with obesity, non-alcoholic fatty liver disease (NAFLD), and aging^2^. Regarding the mechanism of insulin resistance, there are several hypotheses^3^. However, these hypotheses do not cover the role of nervous system in the mechanism^3^. A recent study published in *Cell Metabolism* by Sakamoto et al.^4^ suggests that the sympathetic nervous activity (SNA) plays a crucial role in energy surplus-induced insulin resistance.

IR refers to a pathophysiological state where the body's response or sensitivity to insulin is below the physiological level. In the IR conditions, insulin's activity is reduced in the stimulation of glucose uptake in muscles and adipose tissue, the ability to suppress hepatic glucose output is decreased^5^. Insulin receptor signaling is impaired in the classical theory of IR, while it does not always accompany IR^6^. The role of SNA in IR is controversial, as both increased and decreased SNA have been reported in the literature. The new study found that SNA is increased by energy surplus (nutrition surplus) for IR under challenge with excess-energy, as inhibition of SNA in a transgenic mouse is able to decrease parameters of IR including hyperglucagonemia, adipose tissue dysfunction, and fatty liver disease in the mice^4^. The study provides a set of new evidence regarding SNA in insulin resistance.

The article reports the relationship between SNA and IR by conducting investigation in three model systems. Firstly, excessive activation of the sympathetic nervous system (SNS) was found as a key mechanism leading to IR and metabolic disorders. A high-fat diet (for generation of energy surplus) rapidly increased the levels of norepinephrine (NE) in the plasma of mice, leading to excessive elevation of SNA, and reducing the release of NE from the SNS protected the mice from IR and hyperglycemia. Secondly, lipolysis in adipose tissue (AT) was found as a key mechanism for SNA induction of IR. Excessive activation of the SNS enhanced the activation of hormone-sensitive lipase (HSL) in the white adipose tissue (WAT), promoting lipolysis and resulting in elevated level of plasma glycerol, which induced IR. However, IR was significantly alleviated in adipose tissue-specific ATGL (adipose triglyceride lipase) knockout mice (which regulate lipolysis) under the condition of energy surplus by high-fat diet (HFD). Lastly, using an inducible and peripherally restricted tyrosine hydroxylase (TH; THΔper) knockout mouse model, they found that SNA reduction by gene knockout improved glucose intolerance from short-term and long-term HFD, and improved insulin sensitivity in the liver and AT, while also reduced the risk of fatty liver, inflammation, and adipose tissue fibrosis.

The nerve system regulates the whole body energy metabolism through innervation of peripheral organs or tisuues^7^. The sympathetic nervous releases NE in AT to act on NE receptors on adipocytes, promoting thermogenesis and lipolysis, thereby antagonizes insulin activity in to cause tissue IR. Sensory nerves can transmit leptin signals from AT to the central nervous system^8^. When leptin levels are high, the brain reduces appetite to limit energy intake, thereby improving insulin sensitivity. In the gut, SNS controls blood flow to the intestine to reduce the secretion of digestive enzymes, which slows down the rate of food digestion and glucose absorption, leading to a more gradual insulin secretion and helping to maintain insulin sensitivity. Sensory nerves can sense gut-derived glucagon-like peptide-1 (GLP-1) and transmit satiety signals to the central nervous system, thus limiting food intake. Reduced energy intake can improve insulin sensitivity.

In the pancreas, sympathetic PVN^OXT^ neurons directly regulate insulin secretion. Activation of these neurons reduces insulin secretion and leads to hyperglycemia, while the inhibition induces hypoglycemia^9^. Sensory TRPV1 neurons regulate pancreatic function and systemic glucose homeostasis but intriguingly do not intervene insulin sensitivity. In animal models of type 2 diabetes mellitus (T2DM), such as *db*/*db* mice, three-dimensional panoramic histology revealed an increase in the density of pancreatic sympathetic nerves^10^. Similarly, an increase in islet nerve density has also been observed in the pancreas of T2DM patients, indicating that T2DM is significantly associated with the remodeling of islet nerve input^11^.

In the liver, the metabolic functions are closely controlled by the nervous system^12^. The SNS activate glycogenolysis and gluconeogenesis through secretion of NE, which causes hepatic IR. Sensory nerves, on the other hand, can recognize GLP-1 signal to inhibit hepatic gluconeogenesis for improvement of insulin sensitivity. Subdiaphragmatic vagotomy effectively deactivates vagal nerve signals, inhibits VLDL biogenesis, increases lipid loss in feces, and reduces circulating triglycerides (TG), thereby protecting mice from HFD-induced hepatic steatosis and hyperlipidemia^12^. Yuki Oka and colleagues^13^ have used optogenetic and chemogenetic approaches to precisely activate the CG-SMGRXFP1 neuronal population, which projects to secretory organs such as the liver and pancreas. This activation suppresses bile secretion and stimulates glucagon release. These studies provide more details by which the SNS modulates insulin sensitivity and glucose metabolism through innervation of multiple organs.

In summary, the new study provides a set of convincing evidence to support that SNA is induced by energy surplus in acute manner and chronic manner. More importantly, SNA is opposite to insulin activity leading to IR for induction of energy mobilization and expenditure. Especially, SNA leads to systemic IR through inhibition of the insulin activity in WAT raising glycerol levels in the circulation. This activity is blocked in THΔper mice following inactivation of peripheral SNA by blocking neurotransmitter production. The article has several limitations, mainly that the THΔper model used cannot assess the impact of SNA in specific organs, and further research is needed to determine the roles of systemic and tissue-specific SNA in obesity-related metabolic diseases. In addition, the study was limited to male mice and did not address gender differences and the impact of menopause. In the NE infusion studies, NE levels were outside the physiological range, which may affect the interpretation of the results. Therefore, future research needs to employ more precise models and methods to gain new insight into the SNS roles in obesity and metabolic diseases.

## Author contributions

Shuchou Xia drafted the manuscript. Huili Wu and Jianping Ye provided the idea and revised the manuscript.

## Conflicts of interest

The authors declare no conflicts of interest.

## References

[1] Hill M.A., Yang Y., Zhang L., Sun Z., Jia G., Parrish A.R. (2021). Insulin resistance, cardiovascular stiffening and cardiovascular disease. Metabolism.

[2] Ye J., Yin J. (2024). Type 2 diabetes: a sacrifice program handling energy surplus. Life Metab.

[3] Ye J., Hu Y., Wang C., Lian H., Dong Z. (2023). Cellular mechanism of diabetes remission by bariatric surgery. Trends Endocrinol Metab.

[4] Sakamoto K., Butera M.A., Zhou C., Maurizi G., Chen B., Ling L. (2025). Overnutrition causes insulin resistance and metabolic disorder through increased sympathetic nervous system activity. Cell Metab.

[5] Haeusler R.A., McGraw T.E., Accili D. (2018). Biochemical and cellular properties of insulin receptor signalling. Nat Rev Mol Cel Biol.

[6] Scherer T., Lindtner C., Zielinski E., O'Hare J., Filatova N., Buettner C. (2012). Short term voluntary overfeeding disrupts brain insulin control of adipose tissue lipolysis. J Biol Chem.

[7] Sun M., Wan Y., Shi M., Meng Z.-X., Zeng W. (2023). Neural innervation in adipose tissue, gut, pancreas, and liver. Life Metab.

[8] Caron A., Lee S., Elmquist J.K., Gautron L. (2018). Leptin and brain-adipose crosstalks. Nat Rev Neurosci.

[9] Papazoglou I., Lee J.H., Cui Z., Li C., Fulgenzi G., Bahn Y.J. (2022). A distinct hypothalamus-to-*β* cell circuit modulates insulin secretion. Cel Metab.

[10] Tang S.C., Shen C.N., Lin P.Y., Peng S.J., Chien H.J., Chou Y.H. (2018). Pancreatic neuro-insular network in young mice revealed by 3D panoramic histology. Diabetologia.

[11] Alvarsson A., Jimenez-Gonzalez M., Li R., Rosselot C., Tzavaras N., Wu Z. (2020). A 3D atlas of the dynamic and regional variation of pancreatic innervation in diabetes. Sci Adv.

[12] Khound R., Taher J., Baker C., Adeli K., Su Q. (2017). GLP-1 elicits an intrinsic gut-liver metabolic signal to ameliorate diet-induced VLDL overproduction and insulin resistance. Arterioscler Thromb Vasc Biol.

[13] Wang T., Teng B., Yao D.R., Gao W., Oka Y. (2025). Organ-specific sympathetic innervation defines visceral functions. Nature.

